# Prediction of Strength and Ductility in Partially Recrystallized CoCrFeNiTi_0.2_ High-Entropy Alloy

**DOI:** 10.3390/e21040389

**Published:** 2019-04-11

**Authors:** Hanwen Zhang, Peizhi Liu, Jinxiong Hou, Junwei Qiao, Yucheng Wu

**Affiliations:** 1 College of Materials Science and Engineering, Taiyuan University of Technology, Taiyuan 030024, China; 2 Key Laboratory of Interface Science and Engineering in Advanced Materials, Ministry of Education, Taiyuan University of Technology, Taiyuan 030024, China

**Keywords:** high entropy alloys, precipitation kinetics, strengthening mechanisms, elongation prediction

## Abstract

The mechanical behavior of a partially recrystallized fcc-CoCrFeNiTi_0.2_ high entropy alloys (HEA) is investigated. Temporal evolutions of the morphology, size, and volume fraction of the nanoscaled L1_2_-(Ni,Co)_3_Ti precipitates at 800 °C with various aging time were quantitatively evaluated. The ultimate tensile strength can be greatly improved to ~1200 MPa, accompanied with a tensile elongation of ~20% after precipitation. The temporal exponents for the average size and number density of precipitates reasonably conform the predictions by the PV model. A composite model was proposed to describe the plastic strain of the current HEA. As a consequence, the tensile strength and tensile elongation are well predicted, which is in accord with the experimental results. The present experiment provides a theoretical reference for the strengthening of partially recrystallized single-phase HEAs in the future.

## 1. Introduction

High entropy alloys (HEAs), a new class of structural materials, have attracted a great deal of attention in recent years on account of their special intrinsic characteristics [1,2,3,4,5,6,7,8,9], such as high configuration entropy [10], sluggish atomic diffusion [11], and large lattice distortion [12]. Nevertheless, recent studies indicate that single-phase HEAs, especially single-phase fcc HEAs, the strength is insufficiently for structural applications [13,14]. In other words, strengthening is badly needed so that satisfactory mechanical properties can be achieved. Klimova et al. [15] reported that in the Al- and C-containing CoCrFeNiMn-type high-entropy alloy, substructure strengthening was found to be dominated at low rolling reductions (<40%), while grain (twin) boundary strengthening prevailed at higher strains. Chemical short-range order also has an important influence on the mechanical properties of FeCoNi(AlSi)_*x*_ high entropy alloys [16]. Among the numerous strengthening mechanisms, Precipitation hardening is an effective technique widely used for strengthening high entropy alloys [9,17,18,19,20,21,22]. For example, He et al. [17,23] reported nano-sized L1_2_ coherent precipitates in a face centered-cubic (fcc) NiCoFe alloy with minor additions of Al and Ti, specifically (NiCoFeCr)_94_Ti_2_Al_4_, which exhibits a strength increment of about 327 MPa. It is widely studied that these HEAs are all fully recrystallized, and the grain/grain boundary is thermally stable. In contrast, the partial recrystallized HEAs are rarely studied, the microstructure-property relationship and consequent strengthening mechanism are lacking. In this study, we pay attention to the effect of the partially crystallized microstructure on the mechanical properties. The grain growth mechanisms for the nanoscale precipitation and strengthening mechanisms are fully investigated. In addition, a quantitative model for estimation of tensile ductility is established.

## 2. Materials and Methods 

The mixture of Co, Cr, Fe, Ni, and Ti with purity of at least 99.9% (weight percent, wt.%) was prepared by arc-melting. Under a Ti-gettered argon atmosphere, the sample was cast into an 85 × 10 × 2 mm^3^ copper mould and CoCrFeNiTi_0.2_ (atomic percent, at.%) alloy ingots were prepared. A stable uniform structure was obtained at 1200 °C homogenized for 5 h. The sliced samples were cold-rolled to 75% of the total reduction ratio, and then aged at 800 °C for 3 h, 5 h, 8 h, 10 h, 24 h and 48 h respectively, followed by water quenching. The phase identification was carried out by X-ray diffraction (XRD) using Cu Kα radiation. Then, optical microscopy (OM), scanning electron microscopy (ZEISS SUPRA55 SEM) operated at 20 kV, with the working distance of 9.1 mm, energy dispersive spectrometer (EDS), and JEM-2010 transmission electron microscopy (TEM) were used to observe the surface microstructures. Dog-bone-like tensile specimens with a gauge length of 10 mm, a gauge width of 2 mm and a thickness of 0.5 mm were prepared from aged specimens by electrical discharge machining. Instron 5969 universal testing machine was used to carry out quasi-static tensile tests at room temperature at a constant strain rate of 1 × 10^−3^ s^−1^ greater than or equal to five times.

## 3. Results and Discussion

### 3.1. Precipitation Kinetics

**Figure 1:**
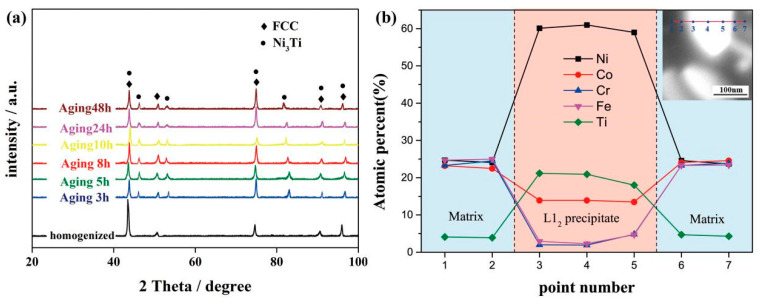
The XRD patterns of the homogenized and the aged alloys (**a**), and EDS-measured composition variation across a single precipitate in the 800 °C/5 h aged alloy(**b**).

**Figure 2:**
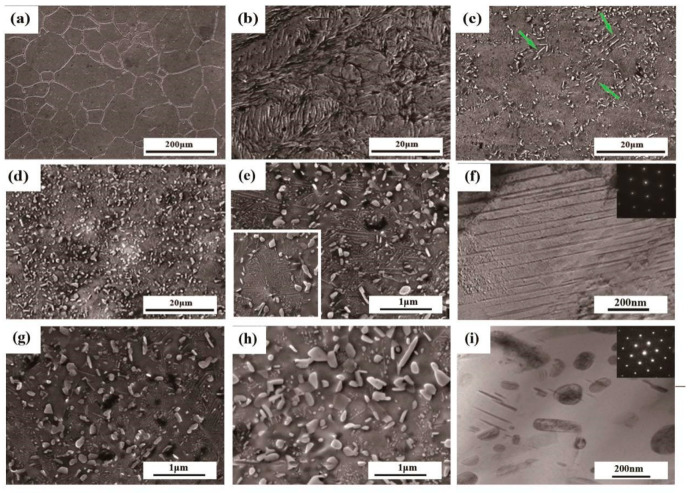
SEM images of the homogenized (**a**), the cold rolled (**b**), the 800 °C/3 h aged (**c–d**) alloys and the aged alloy of different aging time: 5 h (**e**), 10 h (**g**), and 48 h (**h**). The left insert in (**e**) is the corresponding enlarged views of the slip lines in 800 °C/5 h aged alloy. (**f**) and (**i**) show the TEM image of the L1_2_ precipitates and the slip lines in 800 °C/5 h aged alloy, respectively.

As can be seen from Figure 1a, the XRD diagrams of homogenized and aged alloys are displayed. In each alloy, a series of fcc diffraction peaks can be found, which indicates that the matrix of the four alloys is composed of the same fcc phase. In aged samples, an extra series of minor peaks, named L1_2_-Ni_3_Ti, can be detected, which indicates that there is a great probability of the existence of a secondary phase. Figure 2a–e,g–h shows the corresponding SEM micrographs of these HEAs in different statuses. It can be concluded that in Figure 2a, the homogenized alloy demonstrates a single-phase structure, in accordance with the XRD pattern. There are only a few etch pits on the surface probably introduced during electropolishing. After the homogenization process been carried out, the grains are all transformed into equiaxed grains with an average grain size of ~198 μm. Figure 2b exhibits the microstructural feature of the cold-rolled CoCrFeNiTi_0.2_ HEAs. Because of the large CR reduction, there are serious deformation and elongation of grains in the rolling direction, and a high-density of lamellar deformation bands can be dramatically observed after electro-polishing. Similar microstructures have been reported in Fe_34.95_Ni_27.5_Co_17.5_Al_11.5_Cr_8.5_B_0.05_ HEAs [24]. Since the recrystallization temperature of the present HEA is above 800 °C, a large number of slip lines (deformation bands) remain in the matrix. As a consequence, in the recrystallization region, the precipitates spread along the grain boundaries, as shown in Figure 2c. Besides, some plate-shaped precipitates are formed. Generally, upon aging, precipitates often preferentially form at grain boundaries, which is the microstructural heterogeneous site. Moreover, when the aging temperature is low, for instance 800 °C here, the diffusion along the grain boundary is much faster than that in the lattice [23]. This is the main reason why the plate-shaped precipitates grow at grain boundaries. Whereas in the area where the grains are not recrystallized, a great many precipitates are uniformly distributed throughout the matrix, as evidenced in Figure 2d. The measured average compositions of the precipitates and fcc matrix in the 800 °C/5 h aged alloy, as well as the composition variation across a single precipitate is, as shown in Figure 1b. It indicates that the precipitates are enriched with Ni and Ti, but depleted in Co, Cr, and Fe. Meanwhile a part of Ni is substituted by Co. With the above EDS analysis, it is finally identified that the precipitates in the 800 °C/5 h aged alloy is the (Ni,Co)_3_Ti phase. Figure 2i shows the bright field TEM images of aged alloys at 800 °C/5 h. Spheroidal and plate-shaped particles with an average size of 145 nm are found to disperse in a matrix. From the illustration on the right side of Figure 2i, the selected area electron diffraction (SAED) pattern along Z = [011] taken from the precipitates are indexed. The main diffraction points can effectively prove that the matrix does has an fcc structure, whilst additional weak spots observed in the image can also scientifically prove the existence of precipitates with superlattice L1_2_ structure. Han et al. have identified that L1_2_ phase as (Ni,Co)_3_Ti type γ′ phase [25].

As mentioned above, many slip lines remain in the matrix on account of partial recrystallization, which is revealed in Figure 2e. In order to further examine the nature of these slip lines, an enlarged area is selected as exhibited in the left inset of Figure 2e. A significant amount of slip lines along different directions are clearly observed inside the grains. Although the directions of these slip lines are inconsistent, the value of the intersection angle between them is virtually constant as to be about 60°. Figure 2f is the corresponding TEM image of these slip lines in 800 °C/5 h aged alloy. The slip lines are distributed along different directions, and the intersection angle between one another is about 60° as well, which are consistent with SEM images.

**Figure 3:**
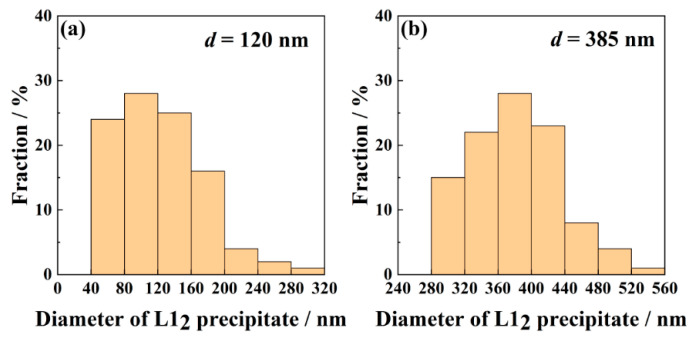
The size distributions of the 800 °C/3 h aged alloy(**a**) and 800 °C/48 h aged alloy(**b**).

To unveil the evolution of precipitation with the time, SEM images for 800 °C/5, 10, and 48 h aged alloys are carefully taken. The precipitates in Figure 2e are basically spheroidal and plate-shaped, uniformly distributed throughout the fcc matrix, the volume fraction is about 15.6 %. As time goes on, the shape of the precipitates turns to be droplet-like and plate-shaped. The average size of precipitates is closely related to the aging time. The longer the aging time is, the larger the average size of precipitates will be. For example, after three hours of aging, the average size of particles is 120 nm, but the average value rises to 385 nm after aging for 48h. The size distributions of the two aged alloys are plotted in Figure 3. Moreover, the precipitate number density *n_v_* is negatively correlated with the aging time. A similar variation of the precipitate with aging time has been reported in many HEAs, such as (NiCoFeCr)_94_Ti_2_Al_4_ [26] and (FeCoNiCr)_100-x-*y*_Ti_*x*_Al_*y*_ [23] HEAs. 

The precipitate number density *n_v_*, was determined from *n_v_* = *n*_a_/*d*, where *n_a_* is the areal density of precipitates measured from SEM micrographs. The precipitate size was defined using an area-equivalent diameter (i.e., diameter = 2$\sqrt{\left(\mathrm{area}⁄\pi \right)}$) measured from SEM micrographs. The average precipitate size *d*, was calculated according to precipitate size distributions. Dividing the areal density by the precipitate diameter to get the number density in three-dimensional space. It should be noted that many slip lines are clearly revealed in the matrix in 800 °C/3 h aged alloy, as shown in Figure 2e. With the aging time, the amount of slip lines is gradually decreased. When the aging time is reached to 48 h, the slip lines do not basically exist in the matrix. This indicates that although the alloy does not fully recrystallize at 800 °C, the recrystallization has been going on with the extension of time.

**Figure 4:**
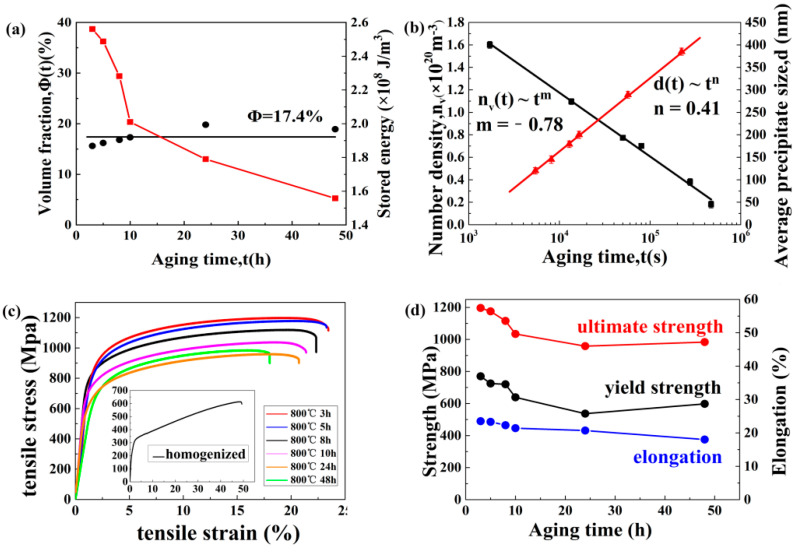
Temporal evolution of (**a**) volume fraction (*Φ*(*t*)) and stored energy (*E*(*t*)), (**b**) number density (*n_v_*(*t*)), and average size (d(t)) of L1_2_ precipitates in the CoCrFeNiTi_0.2_ high entropy alloys (HEA). Tensile stress-strain curves of the homogenized and the annealed alloys(**c**). Detailed tensile strength and the elongation value are plotted in (**d**).

It is very important to study the precipitates growth kinetics in order to further reveal the microstructure-property relationship. The volume fraction (*φ*(*t*)), number density (*n_v_*(*t*)), and average size (*d*(*t*)) are all varies with time, and the relationship is shown in Figure 4a,b, which is corresponding to L1_2_ precipitates in the CoCrFeNiTi_0.2_ HEAs aged at different times in 800 °C. It can be seen from Figure 4a that the value of *φ*(*t*) remains constant when the aging time changed from 3 to 48 h. It is pointed out that the nucleation stage of the precipitation process has been bypassed and entered the coarsening stage directly, therefore *φ*(*t*) remain stable on its equilibrium value (*φ_eq_*) [26]. The stored energy is negatively correlated with the aging time, which is consistent with the theory. With the increase of aging time, the stress concentration is caused by the growth of precipitates, leading to the fracture of the alloy more easily, which is corresponding to the reduction of stored energy. The precipitation number density *n_v_*(*t*) is a function of aging time, as demonstrated in Figure 4b, which indicates the power-law relationship between the two variables at a given temperature. When the aging time is increased from 3 to 48 h, the number density is accordingly decreased from (5.6 ± 0.3) × 10^20^ to (6.4 ± 0.2) × 10^17^ m^−3^. Definitely, the power-law exponent for *n_v_*(*t*), namely the slope of the linear fitted curve is −0.78. Similar temporal exponents also appear in some Ni-Al-based ternary alloys [27,28,29,30]. The size of precipitates also depends on the aging time. It can be concluded from Figure 4b that the average precipitation size *d*(*t*) also follows the power law relationship. The power law index is 0.41.

A model can be used to explain the power-law relationship among the number density, average precipitation size and aging time scientifically and effectively. Philip and Voorhees established the PV model for Ostwald ripening in multi-component systems [31]. According to the model, the coarsening process of precipitates is in accordance with a similar power-law relationship. In other words, the size of precipitates raised to a 1/3 power, due to the increasing aging time. To apply the PV model reasonably to the CoCrFeNiTi_0.2_ HEA, the first step is to concentrate the chemical composition. The baseline of the alloy is fcc-CoCrFeNi. In addition, Ni is the only element with an fcc structure in the alloy, so it can be treated substantially as a Ni-based alloy. Besides that, the lattice constant of CoCrFeNi (0.3572 nm) is highly close to that of Ni (0.3517 nm) [30]. Hence, it seems logical to treat the CoCrFeNiTi_0.2_ as a Ni-based pseudo binary Ni-Ti alloy. Therefore, the PV model of stable coarsening reaction can be used to calculate the coarsening behavior of L1_2_ precipitation in CoCrFeNiTi_0.2_ HEAs [32,33,34,35]:

(1) $$d^{3}\left(t\right)-d^{3}\left(t_{0}\right)=K\left(t-t_{0}\right),$$

(2) $$n_{v}{\left(t\right)}^{-1}-n_{v}{\left(t_{0}\right)}^{-1}=4.74\frac{K}{\varphi_{eq}}\left(t-t_{0}\right),$$

where *K* represents the coarsening rate constant, *d*(*t*), and *n_v_*(*t*) refer to the average size and number density of precipitates at time *t.* Obviously, in Figure 3a,b, the time index for *n_v_*(*t*) is −0.78, and that for *d*(*t*) is 0.41, which is basically consistent with the predicted values of −1 (Equation (3)) and 1/3 (Equation(2)) corresponding to the PV model. The main reason for the slight deviation is probably caused by the fact that the aging time is within a limited duration, but in principle, the coarsening stage of precipitates cannot reach a stable state [36,37,38,39].

### 3.2. Strengthening Mechanisms 

In order to study the effect of microstructure on mechanical properties, uniaxial tensile tests were carried out. As can be seen from Figure 4c, the tensile engineering stress-strain curves of the current seven HEAs under room temperature are measured. The homogenized alloy exhibits high ductility, which reaches 48%, but the yield and ultimate strengths of the alloy are low, which are only 315 and 609 MPa, respectively. A similar value of strength is obtained in CoCrFeMnNi HEAs [1]. Compared to the homogenized alloy, the aged alloys (3 h) exhibit a striking improvement in the mechanical performances, the yield and ultimate strength can reach about ~800 and ~1200 MPa respectively, accompanied by ~20% homogeneous elongation. Detailed tensile strength and the elongation of the aged alloys are plotted in Figure 4d. It is noteworthy that both the tensile strength and elongation are gradually decreased with the aging time. The results can be ascribed to the growing up of the second-phase precipitates with the increasing aging time, which can pin the dislocation movement and accordingly promote the dislocation accumulation, leading to stress concentration, which in turn reduce both the strength and ductility [23].

In precipitation hardening alloys, the particles morphological distribution is the main factor of strength, which covers particle size, particle shape and spacing between particles. The classical Orowan bowing/looping and particles shearing are the main models used to describe the precipitation hardening, which mainly includes. Orowan looping often occurs when the radius of the coherent particles exceeds the critical value or when the particles are incoherent with the matrix. Whereas when the precipitates are small and coherent, the shear mechanism takes place. For the current HEA, the sizes of the precipitates are all more than 100 nm, and the particles are not easily plastically deformed. Therefore, it is concluded that L1_2_ particles strengthen the alloy via Orowan mechanism. The critical stress *σ* or can be expressed in the following [40]:

(3) $$\sigma_{or}=M\cdot \frac{0.4Gb}{\pi \sqrt{1-\upsilon }}\cdot \frac{ln\left(\frac{\bar{d}}{b}\right)}{\lambda },$$

where *M* = 3.06 is the Taylor factor, *G* = 87.5 GPa is the shear modulus, υ = 0.31 is the Poisson ratio, and b = $\sqrt{2}$/2 × $a_{CoCrFeNiTi_{0.2}}$ = 0.255 nm represents the burger vector for an fcc structure. $\bar{d}=\sqrt{2/3}\cdot d$ represents the average precipitate diameter on the slip planes. λ = $\bar{d}$($\sqrt{\pi /(4f)}$−1) represents the average interparticle spacing. Orowan stresses for precipitates in the six aged HEA with different volume fractions and sizes are calculated respectively.

**Figure 5:**
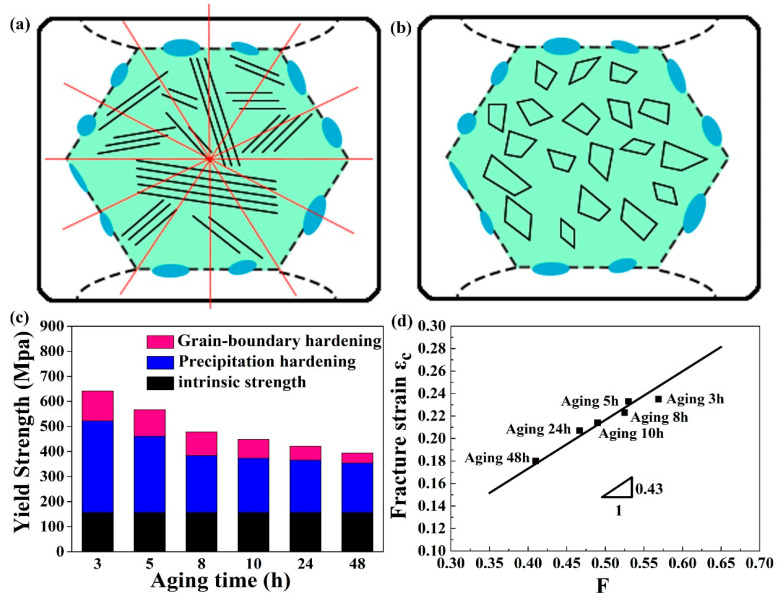
Schematic illustrations showing the slip line be equivalent to sub-grain boundary (**a**) and (**b**). Strengthening contributions from precipitation hardening and grain-boundary hardening in all the aged alloys (**c**). The elongation displayed as a function of the volume fraction and mean particle diameter of L1_2_ phase in the current HEA (**d**).

As mentioned above, a large number of slip lines exist in the alloys along various directions, due to partial recrystallization. These slip lines can be regarded as sub-grain boundaries. Generally speaking, grain refinement can greatly improve the strength of the alloy. The volume fraction of the grain-boundaries is negatively correlated with the grain size. Smaller grain size offers a higher volume fraction of grain-boundaries, which will hinder the movement of dislocations. So here the extensive slip lines can improve the strength by grain-boundary strengthening. To apply the mechanism to the current CoCrFeNiTi_0.2_ HEA, let us consider its equivalent grain size. Figure 5a–b is the corresponding schematic illustration. The slip lines within a grain are selected as the statistical target. Draw twelve lines passing through the center of the grain at 30-degree intervals and measure the length of each line, as Figure 5a shows. Then, it is counted how many segments the 12 lines are divided by the slip lines within a grain and how many segments each slip line within a grain are divided by the 12 lines. Accordingly, it is obtained the length of each line being cut off and the average length. From the two dimensions, the equivalent length of the spacing can be obtained between the slip lines, that is, the average grain diameter. In the statistics at least 50 grains were used for counting in different aging times. In this way, the distance between slip lines within the grains is transformed into the average grain diameter of sub-grain boundaries (Figure 5b). Consequently, the strength increment provided by grain-size refinement can be theoretically predicted.

Hall-Petch equation can explain the relationship between yield strength and grain size scientifically [41]:

(4) $$\sigma_{y}=\sigma_{0}+k_{y}/d^{\frac{1}{2}},$$

where *σ_y_* represents the yield stress, *σ*_0_ represents the lattice friction stress, *k_y_* represents the strengthening coefficient, and d represents the average grain diameter. We can express the increase of yield strength caused by grain size difference ($\Delta \sigma_{G}$) as:

(5) $$\Delta \sigma_{G}=k_{y}\left({d_{p}}^{\frac{-1}{2}}-{d_{A}}^{-\frac{1}{2}}\right).$$

In the formula, *d_P_* denotes the grain size of the thermo-mechanically processed alloys, and *d_A_* represents the grain size of the homogenized alloy.

In order to show the conclusion more intuitively and clearly, the histogram, as shown in Figure 5c, is summarized to explain the strength contribution of the above two strengthening mechanisms. The black part represents the intrinsic strength of the alloy, or the so-called lattice friction strength, according to Moon et al [42], the calculated value is 156 MPa. It can be seen from the diagram that the contribution of precipitation strengthening to strength increment is much greater than that of grain-boundary strengthening. However, with the growth of precipitated particles size, the contribution of precipitation strengthening is gradually decreased, similar to the (FeCoNiCr)_100-*x*-*y*_Ti_*x*_Al_*y*_ HEAs reported before [23]. Moreover, with the aging time, the number of slip lines is decreased, leading to the increased distance between slip lines, thus causing gradually weakened contribution of grain boundary strengthening, which is consistent with the experimental results in Figure 4c.

### 3.3. Prediction of Elongation

Compared with the strength which is relatively easy to evaluate in the existing models, it is quite challenging to quantitatively evaluate the tensile ductility. Aging time is one of the important reasons for the variation of tensile ductility in current HEA. It is due to that with the increase of aging time, the L1_2_ particles grow up gradually, leading to stress concentration, which will lead to a significant reduction in ductility. Here, the present HEAs containing L1_2_ particles can be regarded as ceramic particles-reinforced metal-matrix composites (MMCs) [43]. In discontinuous reinforced metal matrix composites, when the size of the reinforcing phase is micron order and the volume fraction reaches about 20%, the most balanced strength-ductility properties are expected to be exhibited. Similar to metal-based composite alloys, the L1_2_ particles in existing alloys are about 0.1–0.4 μm and the volume fraction is 15.6–19.8%, which appears to be consistent with the model. In order to estimate the elongation of HEAs accurately, a model based on MMCS phenomenologically is adopted [44]:

(6) $$\varepsilon_{c}/\varepsilon_{m}=\left(1-f\right)\left(1+\varepsilon_{cav}\right)\left(1-f_{con}\right),$$

where *ε_c_* represents the failure strain of the composites, *ε_m_* represents the failure strain of the unreinforced matrix, and f represents the volume fraction of particles. In the equation mentioned above, *ε_cav_* = *f *^4/3^/*s* [44] is the contribution to the failure strain from the cavity formation, and *s* represents the aspect ratio of the particles. The parameter *f_con_* in Equation (6) represents the ratio of the constrained to the matrix volume, and can be expressed as [44]:

(7) $$f_{con}=2s/\left[5\left(f^{-1}-1\right)\right].$$

Combining Equations (6) and (7), it is readily obtained

(8) $$\varepsilon_{c}/\varepsilon_{m}=\left(1-f\right)\left(1+\frac{f^{4/3}}{s}\right)\left(1-\frac{2s}{5\left(f^{-1}-1\right)}\right)=F.$$

At present, *ε_c_* can be plotted as a function of *F*, and the result is demonstrated in Figure 4d. It is pointed out that there is a good linear relationship with the slope of the curve to be about 0.43. It can be concluded from the Equation (8) that the slope is predicted to be the fracture strain of the unreinforced alloy matrix, *ε_m_*. Compared with the elongation value of homogenized alloy (~0.49), the value of 0.43 is close to the experimental value. The main reason for the slight offset in Figure 4f is the inhomogeneous distribution of L1_2_ particles with non-random orientation. Nevertheless, it can be concluded from Figure 5d that the tensile ductility in HEA is determined by the volume fraction and morphology of L1_2_ precipitates.

## 4. Conclusions

In this study, a systematic study of the aging behavior of the CoCrFeNiTi_0.2_ HEA is conducted. L1_2_ particles are readily precipitated after annealing at 800 °C for a different time. The temporal exponents for the average size and number density of precipitates are in reasonable accord with the predictions by the PV model for particle coarsening in the current alloys. The ultimate tensile strength can be greatly improved to ~1200 MPa, accompanied with a tensile elongation of ~20% after precipitation. Both grain boundary and precipitates can contribute to strengthening. The tensile strength and tensile elongation are well predicted, which is consistent with the experimental results. The present experiment provides a theoretical reference for the strengthening of partially recrystallized single-phase HEAs in the future.
